# A Predictive Model of Capillary Forces and Contact Diameters between Two Plates Based on Artificial Neural Network

**DOI:** 10.3390/mi14040754

**Published:** 2023-03-29

**Authors:** Congcong Huang, Zenghua Fan, Ming Fan, Zhi Xu, Jun Gao

**Affiliations:** 1 School of Mechanical Engineering, Shandong University of Technology, Zibo 255049, China; 2 Shandong Provincial Key Laboratory of Precision Manufacturing and Non-Traditional Machining, Shandong University of Technology, Zibo 255049, China

**Keywords:** capillary force, liquid bridge, artificial neural network, genetic algorithm, energy minimization

## Abstract

Many efforts have been devoted to the forecasting of the capillary force generated by capillary adsorption between solids, which is fundamental and essential in the fields of micro-object manipulation and particle wetting. In this paper, an artificial neural network (ANN) model optimized by a genetic algorithm (GA-ANN) was proposed to predict the capillary force and contact diameter of the liquid bridge between two plates. The mean square error (*MSE*) and correlation coefficient (*R*^2^) were employed to evaluate the prediction accuracy of the GA-ANN model, theoretical solution method of the Young–Laplace equation and simulation approach based on the minimum energy method. The results showed that the values of *MSE* of capillary force and contact diameter using GA-ANN were 10.3 and 0.0001, respectively. The values of *R*^2^ were 0.9989 and 0.9977 for capillary force and contact diameter in regression analysis, respectively, demonstrating the accuracy of the proposed predictive model. The sensitivity analysis was conducted to investigate the influence of input parameters, including liquid volume and separation distance, on the capillary force and contact diameter. The liquid volume and separation distance played dominant roles in affecting the capillary force and contact diameter.

## 1. Introduction

The liquid bridges have aroused much attention owing to their applicability in industrial applications, including direct scanning probe lithography [1], wafer packaging [2] and 3D printing of electrical circuits [3]. Compared with the volumetric gravitational force, the capillary force generated by the liquid bridge is dominant at the microscopic scale, guaranteeing the feasibility of manipulating micro-components. With the miniaturization trend in the fields of biomedicine [4] and micro-assembly [5], the development of micro-manipulation methods based on the capillary force is significant with some advantages, including great cushioning owing to the liquid, being compatible with complex shapes and self-centering [6].

A microscope 3D structure, “Micro scarecrow”, was constructed using the capillarity of micro-drops [7]. To increase the range of changeable capillary force, Saito et al. [8] designed a concave probe tip, which exhibited a larger pickup ability compared with a flat one. Vasudev et al. [9] developed a microgripper based on electrowetting, which successfully picks up and releases glass beads with a gravity of 77–136 µN by regulating the surface voltage. The liquid meniscus was continuously regulated by pressure adjustment at the capillary nozzle tip to realize capillary force control [10]. Hagiwara et al. [11] designed a gripper that formed water droplets quickly and easily with the movement of a piston slider. The gripper was used to pick up and place several microparts, including a cone, cube and semicylinder, at the submillimeter scale. By controlling the temperature to condense water on the gripper, the condensed droplets were successfully employed to grasp glass beads [12]. Apart from the single microgripper above, multiple-fingers microgrippers were developed with enhanced manipulation capacity. Shigeta et al. [13] proposed a multi-needle gripper that consisted of cylindrically arranged needles. Results were described for the controllable pose of a picked chip resistor. In a further study [14], the orientation of the rectangular micropart was related to the gripper tip shape quantitatively. A double-nozzle gripper was developed to pick up the microparts with different shapes, such as cubes, triangular prisms and helical springs [15].

During the micromanipulation processes, capillary force quantification is critical. The liquid contact area affects the manipulation process of self-alignment of micro parts [16]. Orr et al. [17] solved the Young–Laplace equation using elliptic integrals for an axisymmetric liquid bridge profile between a sphere and a plane. The gravitational influence and the accuracy of the circle approximation of the bridge meniscus shape were evaluated. Willett et al. [18] developed approximations for calculating the capillary force and rupture distance between spheres with equal or unequal radii by numerical integration. The capillary force and rupture distance were studied by three approaches, including numerical solutions of the Young–Laplace equation, ellipse approximation and curve-fitting to the experimental dataset [19]. De Souza et al. [20] investigated the capillary force between plates using different substrate materials to explore the effect of contact angle hysteresis (CAH). The hysteresis of capillary force was directly associated with the measured CAH. Under different cyclic loading speeds, Shi et al. [21] found that a hysteretic loop consisted of four stages, including two pinned (stretching and compression), receding and advancing phases. The advancing angle increased with increasing loading speed while the receding angle decreased. An expression considering the movement of the radial contact line was proposed to describe the correlations. Chen et al. [22] investigated the effect of surface roughness on the liquid bridge shape using the established the volume of fluid model. Theoretical models were constructed based on several assumptions and simplifications, leading to inherent errors between theoretical calculations and experimental values. A predictive model of the capillary force and contact diameter with higher accuracy is needed.

As a powerful data-driven prediction model, artificial neural network (ANN) is proficient in the fitting of non-linear relationships, resulting in extensive applications including solid waste treatment [23], thermal deformation prediction of machine tools [24], axial flow fans optimization [25] and porosity estimation in the geological modeling [26]. Especially in the field of image processing [27,28], ANN was employed as a useful tool. Outputs that were close to real values could be obtained by a well-trained ANN. Ahadian et al. [29] investigated the capillary rise time by employing the ANN model. The results show that the proposed ANN approach could predict the capillary rise time with higher accuracy than the Lucas–Washburn equation. Taghipour-Gorjikolaie et al. [30] used the ANN for the prediction of contact angles and sliding angles on the coated metal surface. It was found that the regression index was 0.9874 for contact angles and 0.992 for sliding angles, exhibiting the high accuracy of ANN prediction. Compared with the results using the response surface methodology method, the constructed ANN model has better accordance with experimental data in terms of optimizing the wettability of rice leaf surfaces [31]. The viscosity and thermal conductivity of various fluids [32,33,34] were predicted by employing the ANN technique.

In this paper, an ANN model optimized by the genetic algorithm (GA) was constructed to predict the capillary force and contact diameter between two plates. A comparison with the theoretical calculation using the shooting method and simulation approach based on the minimum energy method was conducted to verify the accuracy of the ANN model. The effects of input parameters, including liquid volume and separation distance, on the capillary force and contact diameter, were discussed in detail.

## 2. ANN Model and GA Optimization

### 2.1. ANN Model

**Figure 1:**
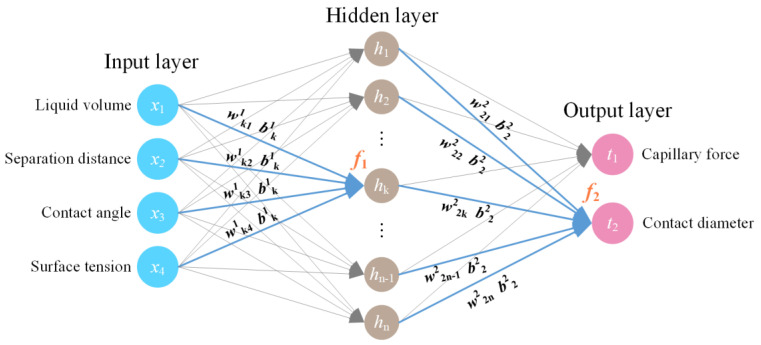
ANN neuronal structure.

Figure 1 shows the constructed three-layer (one input layer, one hidden layer and one output layer) neural structure, the connection between layers and the forward propagation of the ANN. The input layer includes four neurons of liquid volume, separation distance, contact angle and surface tension. The output layer consists of two neurons of capillary force and contact diameter. The number of neurons in hidden layer *n* was determined by the GA optimization.

The activation function of the hidden layer *f*_1_ and the output layer *f*_2_ were defined as follows:

(1) $$f_{1}(x)=\frac{1}{1+e^{-x}},\;f_{2}(x)=x$$

The weights and biases between the input layer and hidden layer and the output of hidden layer *h* were shown.

(2) $$w^{1}=\left[\begin{array}{cccc} w_{11}^{1} & w_{12}^{1} & w_{13}^{1} & w_{14}^{1}\\ w_{21}^{1} & w_{22}^{1} & w_{23}^{1} & w_{24}^{1}\\ \vdots & \vdots & \vdots & \vdots \\ w_{k1}^{1} & w_{k2}^{1} & w_{k3}^{1} & w_{k4}^{1}\\ \vdots & \vdots & \vdots & \vdots \\ w_{n1}^{1} & w_{n2}^{1} & w_{n3}^{1} & w_{n4}^{1} \end{array}\right],\;b^{1}=\left[\begin{array}{c} b_{1}^{1}\\ b_{2}^{1}\\ \vdots \\ b_{k}^{1}\\ \vdots \\ b_{n}^{1} \end{array}\right],\;h=f_{1}(w^{1}\cdot x+b^{1})$$

The weights and biases between the hidden layer and output layer and the output of output layer *t* were

(3) $$w^{2}=\left[\begin{array}{cccccc} w_{11}^{2} & w_{12}^{2} & \cdots & w_{1k}^{2} & \cdots & w_{1n}^{2}\\ w_{21}^{2} & w_{22}^{2} & \cdots & w_{2k}^{2} & \cdots & w_{2n}^{2} \end{array}\right],\;b^{2}=\left[\begin{array}{c} b_{1}^{2}\\ b_{2}^{2} \end{array}\right],\;t=f_{2}(w^{2}\cdot h+b^{2})$$

The initialization method of weights is Glorot uniform distribution [35], defined as

(4) $$G\sim U(-\sqrt{\frac{6}{N_{i}+N_{o}}},\sqrt{\frac{6}{N_{i}+N_{o}}})$$

where *N_i_* is the number of neurons in last layer and *N_o_* is that in next layer. In the present ANN structure: $w^{1}\sim U(-\sqrt{6/17},\;\sqrt{6/17})$ and $w^{2}\sim U(-\sqrt{6/15},\;\sqrt{6/15})$. The biases *b*^1^ and *b*^2^ were initially set to zero.

To judge the difference between predicted values *t* and the actual values *y*, mean square error (*MSE*) was used as the loss function, defined as follows:

(5) $$loss=MSE=\frac{1}{N}\sum_{i=1}^{N}{\left(y_{i}-t_{i}\right)}^{2}$$

where *N* is the number of samples. The difference between *t* and *y* is smaller, as the *MSE* is smaller, indicating the higher accuracy of ANN prediction. 

The correlation coefficient *R*^2^ was used to check the performance of ANN [36]. The accuracy of the predictive outputs is measured by *R*^2^, which is written as

(6) $$R^{2}=1-\sum_{i=1}^{N}{\left(y_{i}-t_{i}\right)}^{2}/\sum_{i=1}^{N}{\left(y_{i}-{\bar{y}}_{i}\right)}^{2}$$

where ${\bar{y}}_{i}$ is the average of actual values. 

To decrease *MSE*, *w* and *b* need to be adjusted to suitable values using a training algorithm. The adaptive moment estimation (Adam) algorithm [37] was employed. Adam algorithm has some advantages, such as easy implementation, high computational efficiency and little memory requirements. Adam algorithm works by dynamically adjusting the learning rate using first-order moment estimation and second-order moment estimation of the gradient.

The first-order partial derivation of *loss* to *w* and *b*, the gradient *g*, is obtained by the back propagation.

The biased first0 and second-moment estimate, *m* and *n*, was updated for timestep *t*:

(7) $$m_{t}=\mu *m_{t-1}+(1-\mu )*g_{t},\;n_{t}=v*n_{t-1}+(1-v)*g_{t}^{2}$$

where *μ* and *v* are the exponential decay rates for the first- and second-moment estimates, respectively. The values of *μ* and *v* are set to 0.9 and 0.999, respectively. 

The bias-corrected moment estimates $\hat{m}$ and $\hat{n}$ were updated as follows:

(8) $${\hat{m}}_{t}=\frac{m_{t}}{1-\mu^{t}},\;{\hat{n}}_{t}=\frac{n_{t}}{1-v^{t}}$$

Large correction is posed to the moment estimates in the initial period. The correction declines with the increasing timestep, which is helpful for convergence. *δ* is updated by the following equation:

(9) $$\delta_{t}=\delta_{t-1}-\alpha *\frac{{\hat{m}}_{t}}{\sqrt{{\hat{n}}_{t}}+\varepsilon }$$

where *α* is the initial learning rate, and *ε* is a small positive number that is set to 10^−7^, preventing the divisor from being 0. The value of *α* is set by the GA. If *α* is too large, the changes in updated parameters will be too great, resulting in the oscillation of *loss*. If *α* is too small, the learning process will be slow. 

The dataset is divided into two parts: the training set and the testing set. The training set is used to train the network (update *w* and *b*), and the testing set is used to test the actual performance of the network (calculate *MSE* and *R*^2^). ANN is trained by a complete dataset once it is termed an epoch. The number of epochs is defined as *ep*. If *ep* is too small, the ANN will not be well trained, leading to a large difference between predictive values and actual values. If *ep* is too large, the network will be overfitted with poor generalization performance, which means the network has good performance on the training dataset while bad performance on the testing dataset. 

An epoch contains several iterations. During an iteration, the number of samples passed to ANN at once is termed the batch size, *bs*. When *bs* is too small, the gradient will change dramatically, causing difficulty in convergence. If *bs* is too large, the convergence will become slow. 

Therefore, the GA is used to determine the four parameters mentioned above (*n*, *α*, *ep* and *bs*). The values of *n*, *α*, *ep* and *bs* were set in the range of 1–20, 0.001–0.1, 500–2000 and 1–102, respectively.

### 2.2. Optimization of ANN Using GA

GA is a stochastic global search optimization algorithm [38], which is employed to optimize the ANN model. The procedures include encoding, decoding, selection, crossover and mutation.

**Figure 2:**
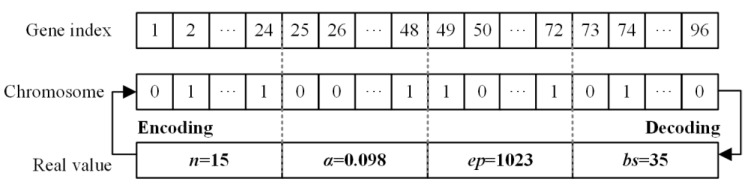
Encoding and decoding procedures.

The mapping from the solution of the problem to the chromosome is called encoding. The inverse transformation is termed decoding. The values of four parameters were all defined by 24-bit binary. These binary strings are combined into a chromosome, representing an individual in the population. The encoding and decoding procedures are shown in Figure 2. If one parameter *δ* ranges from *U*_1_ to *U*_2_, the corresponding decoding equation is

(10) $$\delta =U_{1}+(\sum_{i=1}^{k}b_{i}*2^{i-1})*\frac{U_{2}-U_{1}}{2^{k}-1}$$

where *b_i_* is the *i*-th value of the binary string. For *n*, *ep* and *bs* are integers; the final results of them need to round.

The selection operation is implemented by the roulette wheel algorithm. The minimal *MSE* of the ANN output is considered as the fitness function, which is to measure the merit of individuals in the population. The *MSE* of all individuals constitute set *S*. The probability *p*_k_ that the *k*-th individual is selected is expressed as

(11) $$p_{k}=\frac{s_{\max}-s_{k}+\varepsilon }{\sum_{i=1}^{pop}\left(s_{\max}-s_{i}+\varepsilon \right)}$$

where *s*_max_ is the maximum in *S*, *pop* is the population size and *ε* is set to 10^−4^, preventing *p_k_* from being 0. When the *MSE* of one individual gets smaller, *p_k_* is larger. The number of selections is equal to *pop* to maintain the population, which is set to 30. 

Crossover and mutation operations empower the GA with a local random search capability, which helps to avoid being caught in the local minimum in the evolution processes. The single-point crossover is employed. A crossover point is randomly set in two parental chromosomes. The ligand chromosomes are exchanged with each other from the crossover point to form two new offspring individuals. Two parental chromosomes are randomly paired as well. The new offspring’s chromosomes inherit the characteristic of their parental chromosomes.

**Figure 3:**
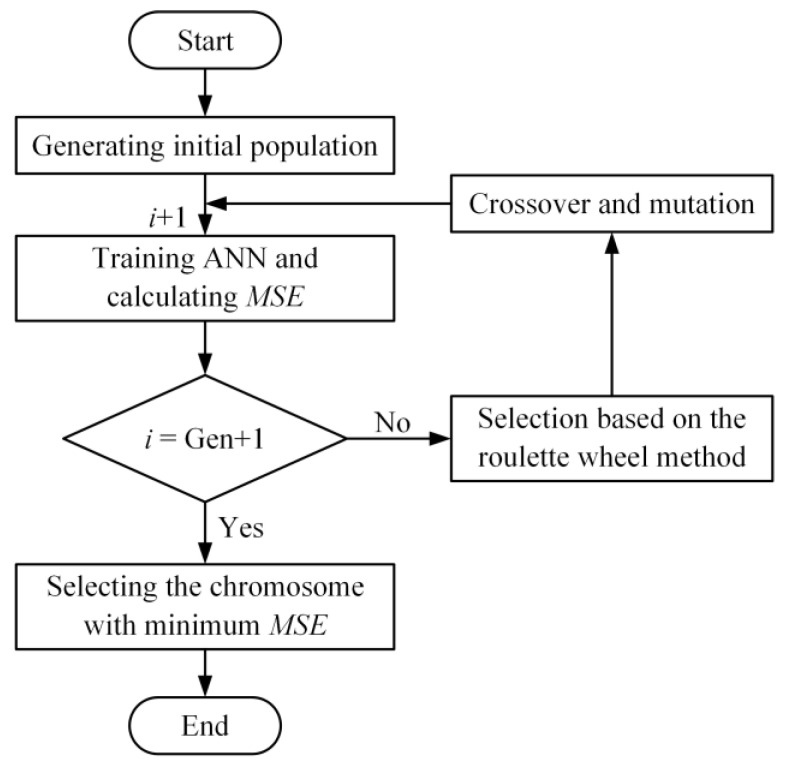
Flowchart of optimizing ANN with the GA.

Mutation introduces innovation in the population and improves the changeability of chromosomes. A single-point mutation is adopted, which works by changing the binary string value from 1 to 0 or from 0 to 1 at a random position on one chromosome. The probabilities of crossover and mutation are set to 0.6 and 0.1, respectively. The processes of optimizing ANN with GA are shown in Figure 3. Python programming language was employed in this study. The main evolution procedures are the following:

Step 1 Generating initial population. The initial population consisting of 20 chromosomes is randomly generated. Each chromosome represents a set of values of *n*, *α*, *ep* and *bs*. The present generation *i* is initially set to 0. The total generation number Gen is set to 100.
Step 2 Training ANN. *i* adds one. ANN is trained and *MSE* is calculated under the condition that each chromosome is represented.
Step 3 Optimization. The processes of selection, crossover and mutation are conducted as *i* is not equal to Gen+1.
Step 4 Generation of the best ANN parameters. Step 2 and step 3 are repeated until *i* is equal to Gen+1. The chromosome with minimum *MSE* is selected as the optimal set of values of *n*, *α*, *ep* and *bs*.

## 3. Experiments

### 3.1. Experimental Setup

**Figure 4:**
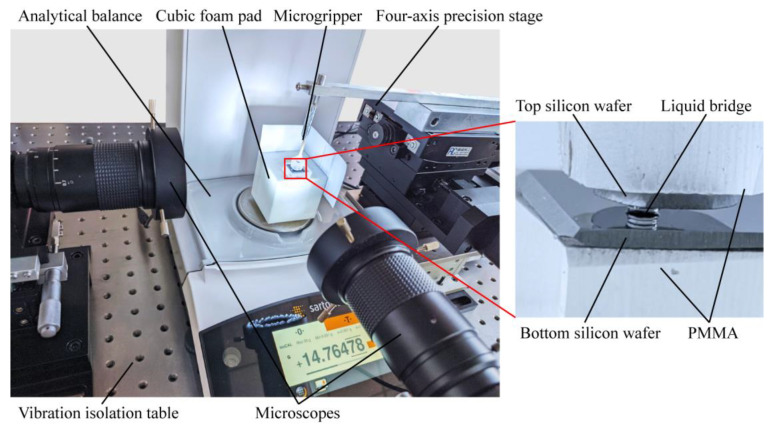
Experimental setup.

An experimental setup was established to control the formation and elongation of a liquid bridge, as shown in Figure 4. The capillary force between two identical silicon wafers was measured using an analytical balance with a resolution of 10^−5^ g. The contact diameter was calculated by processing the liquid bridge images captured by the microscopes. The top silicon wafer and the bottom wafer were attached to the PMMA substrates separately. The top substrate was glued to a single-probe microgripper controlled by a four-axis precision stage with a resolution of 0.125 μm. The bottom substrate was placed on the analytical balance with a cubic foam pad. The instruments above were installed on a vibration isolation table to reduce the vibration transmission.

The properties of liquids, including ethylene glycol (EG), 50 wt% EG and glycerol, were listed in Table 1. *μ*, *γ*, *ρ*, *θ*_s_ and *θ*_r_ is the viscosity, surface tension, density, static angle and receding angle, respectively. *θ*_s_ and *θ*_r_ were calculated using a contact angle goniometer (JC2000D1, POWEREACH). The stretching speed *U* was 10 μm/s. All experiments were conducted at an ambient temperature of 20 ± 2°.

The capillary length *λ*_c_ (defined as $\lambda_{C}=\sqrt{\gamma /\rho g}$), the capillary number *Ca* (defined as Ca=μU/γ), and the Weber number *We* (defined as $We=\rho U^{2}L/\gamma$, where *L* is the characteristic length which was considered to be 1 mm) were calculated, as shown in Table 2. *λ*_c_ was larger than the radius of the droplet used, indicating the surface tension was dominant, and the influence of gravity was negligible. *Ca* and *We* were much less than 1, indicating that the inertial force and viscous force could be neglected [39]. 

The experimental procedure was expounded as follows. A droplet was distributed to the bottom wafer surface with a pipette. The top wafer was controlled to move downward to contact the droplet and stop when a liquid bridge was formed. After a few seconds, the top wafer was moved upward at the speed *U* until the liquid bridge rupture. During the stretching process, the reading of the balance was recorded to reflect the value of capillary force.

### 3.2. Experimental Data

**Figure 5:**
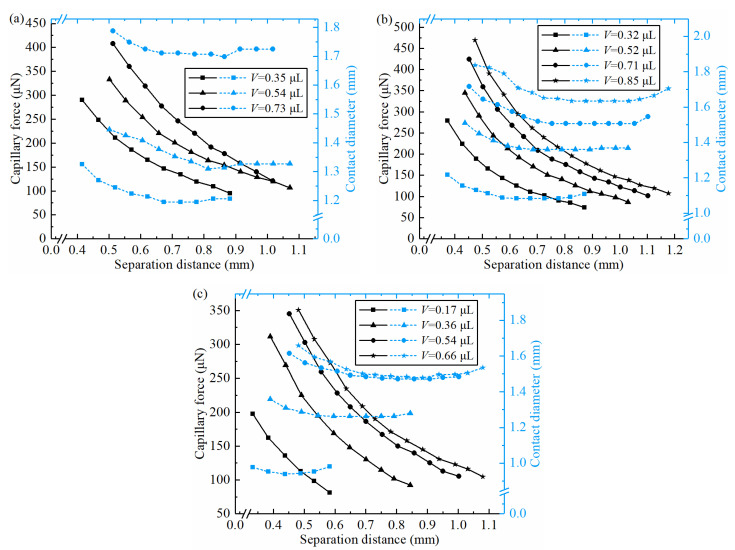
Experimental data: capillary force and contact diameter versus separation distance of different liquids: (**a**) 50 wt% EG, (**b**) EG and (**c**) glycerol.

Liquid volume *V*, separation distance *H*, surface tension *γ* and contact angle *θ* are critical parameters to capillary force *F* and contact diameter *D* in the liquid bridge system. Figure 5 shows the values of *F* and *D* of 50 wt% EG, EG and glycerol, respectively. A total of 128 groups of experimental results were obtained.

## 4. Results and Discussions

### 4.1. ANN Training

A total of 128 groups of experimental data were shuffled and divided into two datasets, 102 for training and 26 for testing. Both the input and the output parameters have been normalized to improve the convergence of ANN:

(12) $$pa=(pa-\bar{X})/X_{\mathrm{std}}$$

where *pa* is a parameter of datasets, $\bar{X}$ is the mean value of datasets and *X*_std_ is the standard deviation of datasets. 

Four parameters of ANN were determined by GA optimization of *n* = 13, *α* = 0.0748, *ep* = 1754 and *bs* = 76, compared with the general values of four parameters of ANN (*n* = 10, *α* = 0.001, *ep* = 2000 and *bs* = 32). The ANN using determined parameters and general parameters were defined as GA-ANN and gANN, respectively.

**Figure 6:**
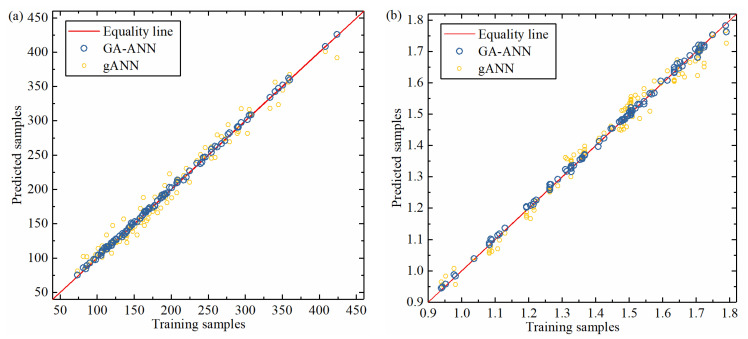
Regression of training dataset: (**a**) capillary force and (**b**) contact diameter.

Figure 6 shows the regression of training dataset for GA-ANN and gANN. The predicted values of GA-ANN exhibited better consistency with the equality line compared with gANN for capillary force and contact diameter shown in Figure 6a,b, respectively. The values of *R*^2^ of capillary force and contact diameter for GA-ANN were 0.9993 and 0.9988, respectively. The *R*^2^ value of capillary force for gANN was 0.9796, and that of contact diameter was 0.9824. Therefore, the GA-ANN model predictions are closer to the real values of the training dataset than the gANN model.

### 4.2. Modeling

#### 4.2.1. Theoretical Model

**Figure 7:**
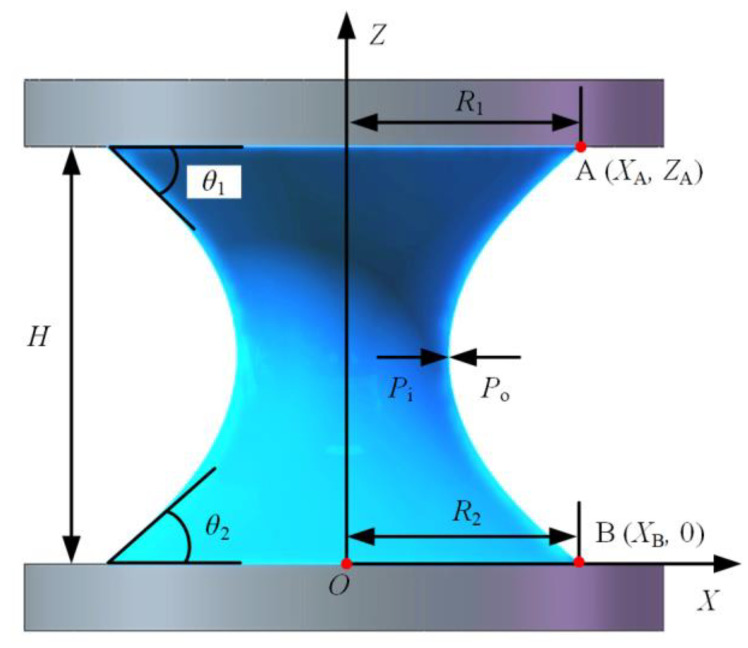
Model of the liquid bridge between plates.

Figure 7 shows the liquid bridge model between two plates without the gravity effect, where *θ*_1_, *θ*_2_ are the contact angles on the top plate and bottom plate, respectively, *H* is the separation distance between plates and *R*_1_ is the contact radius of the liquid bridge on the top plate, *R*_2_ on the bottom plate. The symmetric axis of the liquid bridge is defined as *Z*-axis. The shape of the liquid bridge profile is meniscus due to the pressure difference between the inside liquid pressure (*P*_i_) and the outside air pressure (*P*_o_). The meniscus profile is axisymmetric and expressed by the coordinates (*X*, Z). A (*X*_A_, *Z*_A_) and B (*X*_B_, 0) are the coordinates of nodes where the profile terminates on the top and bottom plates.

The top plate is identical to the bottom plate in the model, so it is deduced that

(13) $$\theta_{1}=\theta_{2},\;R_{1}=R_{2},\;X_{A}=X_{B}$$

The liquid volume *V* is calculated by integration:

(14) $$V=\int_{0}^{Z_{A}}\pi X^{2}dZ$$

The capillary force acting on the bottom plate can be expressed as the sum of the Laplace pressure force *F*_L_ and surface tension force *F*_S_. *F*_L_ derives from the pressure difference, and the vertical component of surface tension force consists of *F*_S_. Thus, the capillary force is given as

(15) $$F=F_{S}+F_{L}=2\pi R_{2}\gamma \sin\theta_{2}+\pi R_{2}{}^{2}\Delta P$$

where Δ*P* is the hydrostatic pressure difference between liquid and air, which is constant at any local point. Based on the built coordinate system, a YoungLaplace equation depicting the profile of the liquid bridge is written as follows:

(16) $$\frac{X^{\prime \prime }}{{\left(1+{X^{\prime }}^{{}^{2}}\right)}^{3/2}}-\frac{1}{X{\left(1+{X^{\prime }}^{{}^{2}}\right)}^{1/2}}=\frac{\Delta P}{\gamma }$$

where $X^{\prime }=dX/dZ$, $X^{\prime \prime }=d^{2}X/dZ^{2}$, *γ* is the surface tension of the liquid. 

To calculate the capillary force, Equation (16) is solved as a two-point boundary value question. Two boundary conditions derived from the nodes on the plates are given as follows:

(17) $$X^{\prime }{|}_{X=X_{A}}=\theta_{1},\;X^{\prime }{|}_{X=X_{B}}=\theta_{2}$$

A shooting method is adopted as follows: (1) Equation (16) is solved numerically with a given *X*_A1_, Δ*P*_1_ and Δ*P*_2_ based on the boundary condition *θ*_1_. Two candidate *θ*_2_ could be obtained. If the target *θ*_2_ is within the range of the two candidates *θ*_2_, Δ*P*_1_ and Δ*P*_2_ will be adjusted to a fixed value based on a dichotomy search method, which will result in a candidate *V*_1_. Similarly, for a given *X*_A2_, a candidate *V*_2_ can be obtained. If the target *V* is within the range of two candidates *V*, the profile of the liquid bridge would be further adjusted to reach the target *V*. *X*_B_ is obtained in the solution process and compared with *X*_A_. If *X*_B_ is not equal to *X*_A_, it indicates that the solution is non-stable; otherwise, the solution is the stable and correct solution.

#### 4.2.2. Simulation Model

An alternative way was adopted to calculate the capillary force and contact diameter based on the minimum energy method. The simulation is conducted using the software package Surface Evolver (SE). The gravity effect was not considered in the simulation model. The total energy of a liquid bridge system consists of three parts: the solid–liquid (*A*_sl_*γ*_sl_), the solid–gas (*A*_sg_*γ*_sg_) and the liquid–gas (*A*_lg_*γ*_lg_) interfacial energies, where *A* and *γ* are the area and surface tension of the interface, respectively. Thus, the total interfacial energy *E* of the liquid bridge system is expressed as

(18) $$E=A_{\mathrm{sl}}\gamma_{\mathrm{sl}}+A_{\mathrm{sg}}\gamma_{\mathrm{sg}}+A_{\mathrm{lg}}\gamma_{\mathrm{lg}}$$

**Figure 8:**
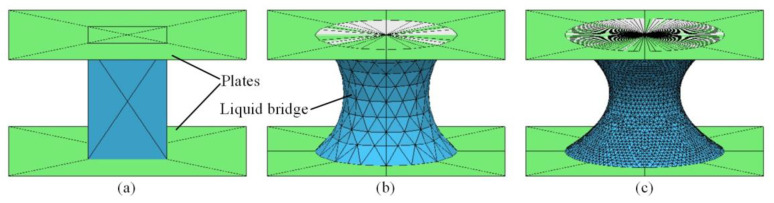
Surface evolution of the liquid bridge system: (**a**) initial definition, (**b**) deformation of evolution and (**c**) finished evolution with a stable liquid bridge.

Figure 8 shows the evolution processes of the liquid bridge between plates. In the SE model, a finite-element method is employed. The surfaces of the liquid bridge and top and bottom plates are represented as a mesh of triangles, which were connected in an arbitrary topology, as depicted in Figure 8a. Toward the goal of minimum total energy, the capillary bridge deformed and evolved by obeying several constraints, including constant volume and contact angles, as shown in Figure 8b. The stopping criteria for evolution is set as the absolute difference of *E* between two adjacent evolutions is smaller than 10^−6^. Figure 8c shows that a stable liquid bridge with minimal energy is established.

The capillary force *F* generated by the liquid bridge is calculated as

(19) $$F=\gamma_{\mathrm{lg}}l\sin\theta_{2}-\Delta pA_{b}$$

where Δ*p*, *l* and *A*_*b*_ are the pressure difference, contact line length and the contact area of the liquid bridge on the bottom plate, respectively. Their values can be obtained from the evolved SE model, as well as the contact diameter.

### 4.3. Comparison of GA-ANN, gANN, Simulation and Theoretical Solutions

**Figure 9:**
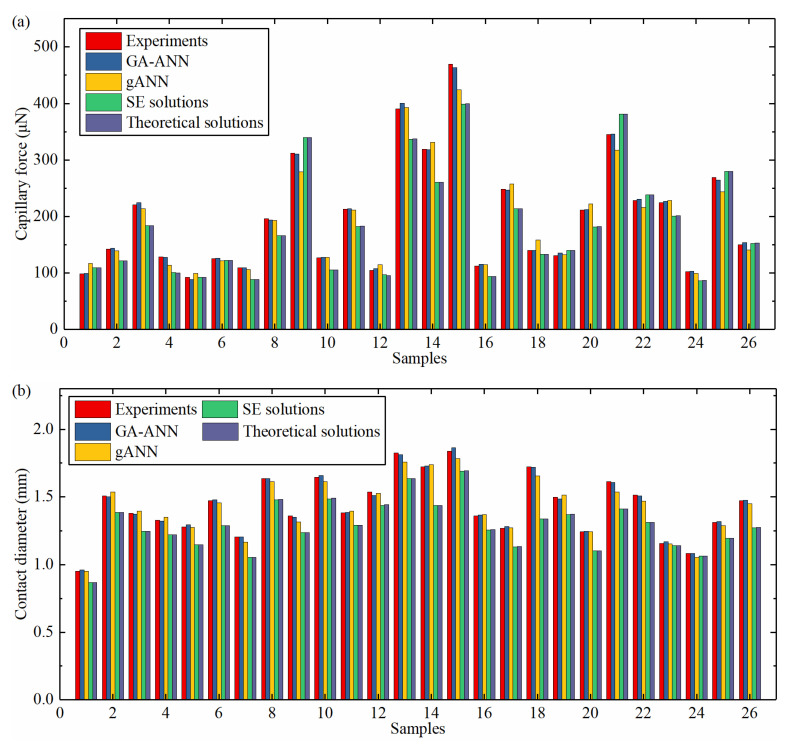
Comparison of prediction results: (**a**) capillary force and (**b**) contact diameter.

Figure 9 shows the predicted values by GA-ANN, gANN, simulation and theoretical solutions. The contact angle used in the SE and theoretical solution methods are receding angle *θ*_r_ because of contact angle hysteresis (CAH) [20]. In general, the predicted capillary force by the four methods exhibited good agreement with the experimental values on the 27 testing samples, as shown in Figure 9a. All four methods can be used to predict the capillary force with good accuracy. 

Predictions of GA-ANN match the experimental results more closely than that of gANN. Table 3 shows that the *MSE* values of capillary force are 10.3 and 244.706 for GA-ANN and gANN, respectively. It indicates that the ANN is a promising method to predict the capillary force. The ANN model trained by GA has a better predictive ability than the general one.

Compared with the ANN predictions, the results of SE and theoretical solutions exhibit a larger difference from the experiments. The *MSE* of capillary force by SE solution is 865.883, while that by theoretical solution is 860.581. The difference between predictions and experimental values can be explained by the error in contact angles used between the constructed models and the real liquid bridge. In SE and theoretical models, the contact angles are set to a constant, while the contact angles are varied with the stretching process of the liquid bridge [40]. Additionally, the results of SE solutions are consistent well with theoretical solutions, as demonstrated in reference [41].

Predictions of the GA-ANN, gANN, SE and theoretical solutions in terms of contact diameter are plotted in Figure 9b. ANN methods (GA-ANN and gANN) show good consistency with experimental values. However, contact diameter values predicted by SE and theoretical solutions exhibit large errors with experimental values. That is foreseeable due to the effect of CAH. By calculating the *MSE* of contact diameter, the GA-ANN is proved to be the most accurate predictive method of the four methods, and the value of *MSE* is 0.0001. This indicates the powerful prediction capability of ANN and the effectiveness of GA optimization.

**Figure 10:**
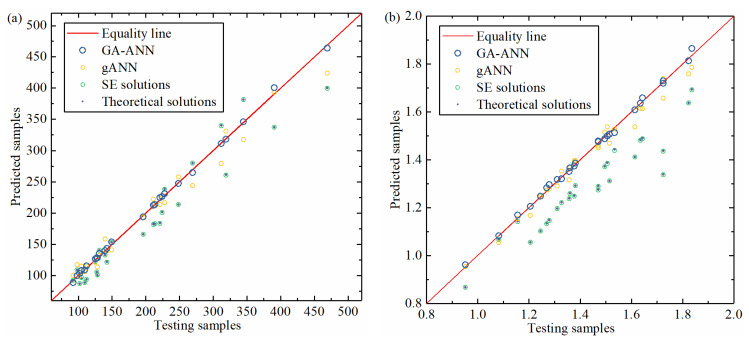
Comparison in regression: (**a**) capillary force and (**b**) contact diameter.

In Figure 10, the regression comparison between GA-ANN, gANN, SE and theoretical solutions is presented. When *R*^2^ gets closer to 1, the prediction gets more accurate. *R*^2^ of capillary force for GA-ANN, SE and theoretical solutions are 0.9989, 0.9109 and 0.9114, respectively. In terms of contact diameter, *R*^2^ of SE and theoretical solutions are 0.4389 and 0.4468, respectively, indicating SE and theoretical solution methods are not suitable for predicting contact diameter. Inversely, *R*^2^ of contact diameter for GA-ANN is 0.9977, which shows the strong prediction ability of ANN. 

### 4.4. Effects of Input Parameters on Capillary Force and Contact Diameter

**Figure 11:**
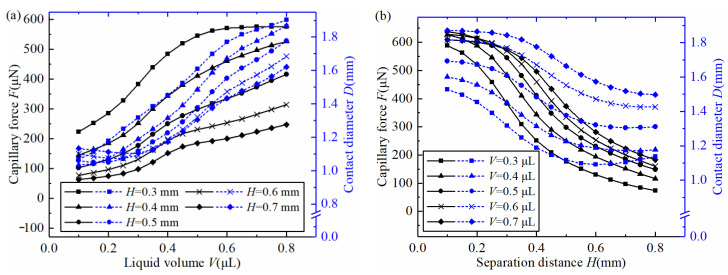
Capillary force and contact diameter versus input parameters: (**a**) changing *V* with various *H* (*θ* = 36°, *γ* = 50 mN/m), (**b**) changing *H* with various *V* (*θ* = 36°, *γ* = 50 mN/m).

The trends of capillary force *F* and contact diameter *D* with liquid volume *V*, separation distance *H* are depicted in Figure 11. All data were obtained from the constructed GA-ANN model. The values of *F* increase with ascending *V*, whereas the opposite impact appears along *H*. The effect of *V* is mainly due to the fact that *D* gets larger with increasing *V*, which results in ascending *F* according to the trend of *D* in Figure 11a and Equation (16). Likewise, the separation distance gets larger, leading to the decrease of *D*, and *F* gets smaller eventually, as shown in Figure 11b. The prediction results by GA-ANN are consistent with theoretical analysis.

Sensitivity analysis is conducted to quantitively evaluate the effect of input parameters based on the connection weight approach [31]. The influence of parameters in the model is judged by calculating the products of input–hidden weight and hidden–output weight and summing them, which is defined as

(20) $$S_{i,k}=\sum_{j=1}^{n}w_{ij}^{1}w_{jk}^{2}$$

The percentage of the absolute sums is calculated as

(21) $$p=\frac{\left|S_{i,k}\right|}{\sum_{k=1}^{4}\left|S_{i,k}\right|}\times 100\%$$

Table 4 shows the values of the weight of the constructed ANN model. The results show that liquid volume and separation distance dominate the outputs rather than the contact angle and surface tension. For capillary force, the effect of liquid volume is the most significant, while for contact diameter, the separation distance is dominant.

## 5. Conclusions

In this paper, an artificial neural network (ANN) model with three layers was developed to predict the capillary force and contact diameter of the liquid bridge between two plates. Four parameters, including liquid volume, separation distance, contact angle and surface tension, were employed as input parameters of the ANN model. The optimal ANN parameters determined by the genetic algorithm (GA-ANN) were that the number of hidden layer neurons was 13, the learning rate was 0.0748, the number of epochs was 1754, and the batch size was 76. Compared with the theoretical solution method of the Young–Laplace equation and simulation approach based on the minimum energy method, the ANN prediction model was more accurate by calculating the mean square error (*MSE*) and correlation coefficient (*R*^2^). In terms of GA-ANN, the *MSE* of the capillary force and contact diameter was 10.3 and 0.0001, respectively. The regression analysis showed that for GA-ANN, *R*^2^ of the capillary force was 0.9989, and that of the contact diameter was 0.9977. The sensitivity analysis showed that the capillary force was subject to liquid volume while the contact diameter was subject to the separation distance. The developed ANN model enabled the precise prediction of the capillary force and contact diameter, providing a powerful tool for studying liquid bridges. 

## Figures and Tables

**Table 1:** Experimental liquids and physical properties.

Liquids	*μ* (Pa s)	*γ* (mN/m)	*ρ* (g/cm^3^)	*θ*_s_ (°)	*θ*_r_ (°)
Ethylene glycol	0.021	48.4	1.11	41.7	34.4
50 wt% ethylene glycol	0.004	57	1.07	50.3	40.4
Glycerol	0.243	63.4	1.26	42.6	33.3

**Table 2:** Rheological properties of experimental liquids.

Liquids	*λ*_c_ (mm)	*Ca*	*We*
Ethylene glycol	2.109	4.34 × 10^−6^	5.28 × 10^−4^
50 wt% ethylene glycol	2.331	7.02 × 10^−7^	2.67 × 10^−3^
Glycerol	2.266	3.83 × 10^−5^	5.18 × 10^−5^

**Table 3:** Values of *MSE* and *R*^2^ for different models.

Models	*MSE* of Capillary Force	*MSE* of Contact Diameter	*R*^2^ of Capillary Force	*R*^2^ of Contact Diameter
GA-ANN	10.3	0.0001	0.9989	0.9977
gANN	244.706	0.0011	0.9748	0.9764
SE solutions	865.883	0.0268	0.9109	0.4389
Theoretical solutions	860.581	0.0265	0.9114	0.4468

**Table 4:** Weights of GA-ANN.

Neuron Number	Weight					
	Liquid Volume (*x*_1_)	Separation Distance (*x*_2_)	Contact Angle (*x*_3_)	Surface Tension (*x*_4_)	Capillary Force (*t*_1_)	Contact Diameter (*t*_2_)
1	0.272	2.657	3.642	−1.608	−0.34	1.218
2	−0.234	−3.530	−0.280	1.944	0.073	−1.300
3	0.702	−1.779	1.869	−1.682	0.450	−0.802
4	1.818	−1.430	0.163	−1.043	−0.801	0.219
5	1.769	−1.570	−0.290	−0.597	−0.060	0.425
6	−3.487	1.240	−0.307	0.159	2.647	0.817
7	−2.176	1.248	0.141	0.214	2.013	1.052
8	2.566	−4.556	0.490	1.668	0.039	−0.464
9	1.481	−1.650	0.520	1.775	−0.811	0.865
10	0.593	2.880	−0.978	−1.805	−0.639	−0.288
11	0.978	−4.842	−0.817	−0.700	−0.909	−0.123
12	−1.150	2.577	0.790	−0.118	0.489	0.409
13	0.614	−0.858	3.061	−1.881	−0.120	−0.636
Sum of products for capillary force	−17.970	10.159	−0.076	2.239	-	-
Importance of capillary force	1	2	4	3	-	-
Percentage	59.0%	33.3%	2.4%	7.3%	-	-
Sum of products for contact diameter	−4.975	12.646	2.093	−0.747	-	-
Importance of contact diameter	2	1	3	4	-	-
Percentage	24.3%	61.8%	10.2%	3.7%	-	-

## Data Availability

The data presented in this study are available on request from the corresponding author.

## Nomenclature

**Symbol**	**Name**
*n*	Number of neurons in the hidden layer
*f*	Activation function
*w*	Weight
*b*	Bias
*h*	Output of hidden layer
*t*	Predicted value of ANN
*y*	Actual value of dataset
*R*_2_	Correlation coefficient
*α*	Initial learning rate
*ep*	Number of epochs
*bs*	Number of samples passed to ANN at once
*p*	Selected probability in the GA optimization process
*pop*	Population size in GA
Gen	Total generation number
*μ*	Viscosity
*γ*	Surface tension
*ρ*	Density
*θ*_s_	Static contact angle
*θ*_r_	Receding contact angle
*U*	Stretching speed
*λ*_c_	Capillary length
*L*	Characteristic length of system
*V*	Liquid volume
*H*	Separation distance
*F*	Capillary force
*D*	Contact diameter
GA-ANN	ANN optimized by GA
gANN	ANN employing general parameters
**Abbreviation**	**Name**
CAH	Contact angle hysteresis
ANN	Artificial neural network
GA	Genetic algorithm
*MSE*	Mean square error
EG	Ethylene Glycol
*Ca*	Capillary number
*We*	Weber number
